# The *Drosophila melanogaster* Seminal Fluid Protease “Seminase” Regulates Proteolytic and Post-Mating Reproductive Processes

**DOI:** 10.1371/journal.pgen.1002435

**Published:** 2012-01-12

**Authors:** Brooke A. LaFlamme, K. Ravi Ram, Mariana F. Wolfner

**Affiliations:** 1 Department of Molecular Biology and Genetics, Cornell University, Ithaca, New York, United States of America; 2 Embryotoxicology Division, Council of Scientific and Industrial Research–Indian Institute of Toxicology Research, Lucknow, India; University of Arizona, United States of America

## Abstract

Proteases and protease inhibitors have been identified in the ejaculates of animal taxa ranging from invertebrates to mammals and form a major protein class among *Drosophila melanogaster* seminal fluid proteins (SFPs). Other than a single protease cascade in mammals that regulates seminal clot liquefaction, no proteolytic cascades (i.e. pathways with at least two proteases acting in sequence) have been identified in seminal fluids. In *Drosophila*, SFPs are transferred to females during mating and, together with sperm, are necessary for the many post-mating responses elicited in females. Though several SFPs are proteolytically cleaved either during or after mating, virtually nothing is known about the proteases involved in these cleavage events or the physiological consequences of proteolytic activity in the seminal fluid on the female. Here, we present evidence that a protease cascade acts in the seminal fluid of *Drosophila* during and after mating. Using RNAi to knock down expression of the SFP CG10586, a predicted serine protease, we show that it acts upstream of the SFP CG11864, a predicted astacin protease, to process SFPs involved in ovulation and sperm entry into storage. We also show that knockdown of CG10586 leads to lower levels of egg laying, higher rates of sexual receptivity to subsequent males, and abnormal sperm usage patterns, processes that are independent of CG11864. The long-term phenotypes of females mated to CG10586 knockdown males are similar to those of females that fail to store sex peptide, an important elicitor of long-term post-mating responses, and indicate a role for CG10586 in regulating sex peptide. These results point to an important role for proteolysis among insect SFPs and suggest that protease cascades may be a mechanism for precise temporal regulation of multiple post-mating responses in females.

## Author Summary

Proteases can destroy, activate, or otherwise modulate the function of other proteins. In seminal fluid, many proteins have to be activated or degraded after mating; proteolysis is an effective way to accomplish this because seminal fluid proteins act outside of the cell, where most other regulatory processes cannot be used. Despite the presence of proteases in the seminal fluid of many animals, nearly nothing is known about the kinds of processes they regulate. Here, we present evidence of a protease cascade in the seminal fluid of the fruit fly *Drosophila melanogaster*. This cascade involves two proteases that are activated during mating. Once in the female, the downstream protease acts on two other proteins that are important for ovulation and sperm storage. Interestingly, the protease at the top of the cascade, CG10586, is also required for other female post-mating responses, including egg laying and sperm usage, independent of the second protease. Thus, CG10586 might be a general regulatory switch used by the male to quickly activate many female responses after mating.

## Introduction

Proteolysis regulators are a component of the seminal fluid of many animal taxa, including insects and other invertebrates [1]–[7], fish [8]–[10], birds [11], [12], and mammals [13]–[18]. However, the mechanisms by which seminal proteases act, and most of the processes they affect, in mated females are poorly understood.

A mechanism by which proteases may effect physiological responses is through proteolytic cascades. Because most proteases are synthesized as inactive zymogens and require the removal of a short N-terminal sequence for activation [19], a protease cascade can be rapidly set in motion without new protein synthesis. For example, in mammals a seminal protease cascade activates the protease prostate specific antigen (PSA), in order to rapidly liquefy the seminal clot formed following ejaculation (reviewed in [15]). The action of PSA is regulated, in part, by the protease inhibitor PCI (reviewed in [20]), which controls the timing and extent of liquefaction. Seminal clots are an important feature of the post-mating response in many animals [13], [21], [22].

Given the prevalence of proteolysis regulators in seminal fluid, it seems likely that they are involved in other processes whose effects may extend past the first few minutes after mating. The study of seminal fluid protease functions would benefit greatly from a genetic approach. *Drosophila melanogaster* provides an excellent system in which to study the roles of seminal fluid proteolytic proteins. Analysis of *Drosophila* seminal fluid proteins (SFPs) capitalizes on a wide range of available genetic tools, physiological and behavioral assays, and both a well-annotated genome and seminal fluid proteome. In addition, though individual SFPs, including proteases [23], are not generally well-conserved between distant taxa [24], [25], the biochemical classes into which SFPs fall are conserved between insects and mammals [21], [26], suggesting that mechanisms of action are likely to be conserved as well.

Approximately 18% of the proteins in the *Drosophila* ejaculate have been identified as predicted proteases or protease inhibitors [2], [27]. Mass spectrometry-based estimates indicate that the abundance of individual proteolysis regulators varies, with some being the most abundant proteins in the ejaculate (*e.g.* Acp62F) and others being the least abundant (*e.g.* CG10587) [2]. Most SFP predicted proteolysis regulators are either serine proteases or serine protease inhibitors with unknown functions [2], [28], [29], though a few other protease classes have also been identified [26], [30]–[32]. Proteolysis regulators have been identified as expressed in male reproductive tract tissues of *Tribolium* [7] and directly in the ejaculates of honey bees [3] and mosquitoes [33]. In crickets, a predicted trypsin-like serine protease in the ejaculate is important for inducing egg laying in mated females [5]. In the nematode *Caenorhabditis elegans*, a trypsin SFP has recently been reported to function in activation of male sperm [10]. Though proteases are emerging as a common SFP class in animals, there have been no studies determining whether protease cascades (*i.e.* proteolytic pathways that require at least two proteases in sequence) are a common regulatory mechanism for seminal fluid-mediated post-mating traits.

In *Drosophila*, transfer of SFPs from male to female during mating induces physiological changes in mated females [reviewed in 6]. Two of these changes are increased egg production and reduced receptivity to remating. These changes occur in two phases: short-term and long-term, both of which are necessary for optimal fertility. The short-term response (STR) occurs within 24 hours of mating and is solely dependent on the receipt of SFPs [34], including the prohormone ovulin [35], CG33943 [36], the sperm storage protein Acp36DE [37], [38], and the action of free sex peptide (SP) that is not bound to sperm [39], [40].

Long-term persistence of post-mating changes (the long-term response, or LTR) requires SP and multiple other SFPs, and the presence of sperm in storage [40]. SP binds to sperm during mating. Cleavage by an unknown trypsin protease(s) is required to release the active portion of SP from sperm within the mated female [41]. SP is gradually cleaved from stored sperm during the approximately two weeks that they remain in storage. As long as SP is released into the female, she continues to lay eggs at a high rate and is more likely to reject courting males [41]. If SP cannot be released from sperm, the LTR does not occur [41].

Fertility defects arise if SP cannot bind to sperm in the mated female, or if it cannot be released from sperm. Sperm binding by SP requires the action of at least four other SFPs: the predicted serine protease CG9997, the Cysteine Rich Secretory Protein (CRISP) CG17575, and the gene duplicate pair lectins CG1652 and CG1656 [36], [42]. These four “LTR proteins”, together with SP, function in an interdependent network to bind SP to sperm as well as to localize each other to the seminal receptacle (SR), the major sperm storage organ of the female [42]. In this network, CG9997 is cleaved into a 36-kDa protein in the male ejaculatory duct/bulb, prior to transfer to the female and is required for the normal transfer of CG1652 and CG1656. CG17575 is required to localize CG1652 and CG1656 to sperm and the SR. This final step is then required for SP to bind sperm and accumulate in the SR. If any one of the four LTR proteins is absent, SP does not bind sperm. These SP-free sperm are still stored in normal numbers, but cannot be efficiently released from storage for fertilization past the first 24 hours after mating [42], because SP is also required for sperm release [43].

In addition to SP, two SFPs involved in post-mating traits are known to be cleaved following deposition into the female. The prohormone ovulin is initially cleaved at about 10 minutes after the start of mating (ASM) [44]. Ovulin is required for a maximal ovulation rate in the first 24 hours following mating [35], [45]. Processing occurs via three cleavage events from the N-terminus of ovulin that ultimately results in the production of one major cleavage product (approx. 25 kDa) and three minor products (each 5 kDa or smaller) [44]. Ectopic expression experiments have shown that both full-length ovulin as well as two C-terminal fragments, roughly corresponding to cleavage products of ovulin, are each able to independently induce ovulation in virgin females [46]. The glycoprotein Acp36DE is also cleaved within mated females [47], starting at approximately 20 minutes ASM, as detected by Western blot [47], [48]. Acp36DE is required for efficient sperm storage [37], [48]. This protein is responsible for the conformational changes of the uterus immediately following the start of copulation, which are thought to aid the movement of sperm into the storage organs [38], [49].

A previous study of 11 SFP proteases and protease inhibitors identified CG11864 as required for processing of both ovulin and Acp36DE [32]. Though all three proteins are produced in the male accessory glands, ovulin and Acp36DE are not cleaved until several minutes after their entry into the female reproductive tract. Therefore, three possibilities exist for the regulation of ovulin and Acp36DE cleavage. CG11864 may be activated during mating, a repressor of CG11864 activity may be removed during mating, or a combination of both may occur.

CG11864 is predicted to be a member of the astacin family of metalloproteases, based on sequence similarity [32]. Astacin family proteases, like many other proteases, require removal of an N-terminal pro-peptide for activation [50]. The activity of CG11864 thus may be regulated in a similar manner. CG11864 is produced in the male accessory glands as a 33-kDa protein and is cleaved to an approximately 30-kDa form [32]. This cleavage begins in the male reproductive tract, in the ejaculatory duct and/or bulb, while CG11864 is in transit to the female during mating [32]. The size of the cleaved form of CG11864 is consistent with removal of a predicted pro-peptide from the N-terminus. We hypothesize that cleavage of CG11864 is required for its activation. If this is the case, there should be factors produced by the male that regulate the activation of CG11864. However, the previous study involving 11 SFP proteolysis regulators did not suggest their requirement for the regulation of CG11864 [32]. A recent microarray analysis by Chintapalli et al. [51] and subsequent proteomic studies by Findlay et al. [2] identified additional serine proteases in the ejaculate. We, therefore, focused on these proteases to test for roles in the activation/regulation of seminal proteolysis.

Here, we used RNAi knockdown analysis to test five male-derived serine proteases for roles in ovulin cleavage and other reproductive events. We describe the first proteolytic cascade in fly seminal fluid that is regulated by a predicted trypsin-like serine protease, CG10586. We propose to rename this enzyme seminase (gene symbol: *sems*). Seminase is required for cleavage, and likely activation, of CG11864. Like CG11864, seminase is produced in the accessory glands and is cleaved in the male during copulation. We show that CG11864 is not able to undergo self-cleavage in the absence of seminase. In addition to regulating CG11864 and thus its downstream SFP substrates, we show that seminase is a member of the LTR network, a CG11864-independent pathway that results in SP binding to sperm.

## Results

### Seminase Is Required for Normal CG11864-Mediated Processing of Ovulin and Acp36DE

**Figure 1:**
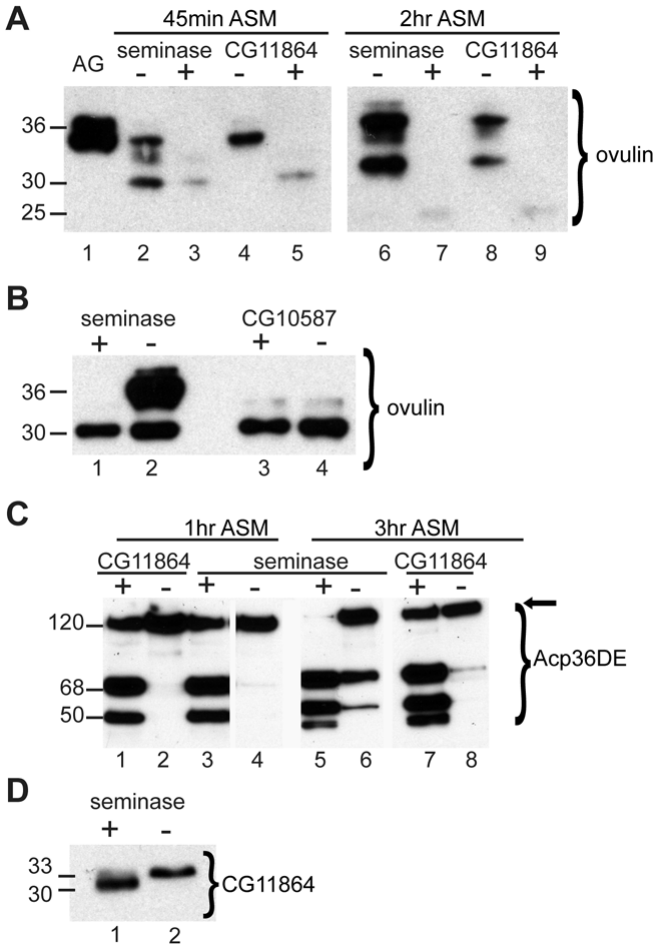
Processing of SFPs is defective in the absence of seminase. (A) Western blot probed with ovulin antibody. Lane 1: full-length ovulin in male accessory glands (AG). Lanes 2–9: female reproductive tracts (RT) dissected after mating to control (+) or RNAi (−) males for the gene given above the lanes. Females were dissected at 45 minutes after the start of mating (ASM) (lanes 2–5) or 2 hours ASM (lanes 6–9). All lanes are from the same gel with extraneous lanes removed for clarity. (B) Western blot probed with ovulin antibody. All lanes are female RT dissected at 30 minutes ASM. Lanes 1 and 2 are from females mated to seminase control (+) or RNAi (−) males. Lanes 3 and 4 are from females mated to CG10587 control (+) or RNAi (−) males. (C) Western blot probed with Acp36DE antibody. All lanes are from the same gel, with extraneous lanes cut out for clarity. Lanes 1–8 contain RT from females mated to males of the given genotype as in (A). Females were dissected at 1 hour ASM (lanes 2–5) or 3 hours ASM (lanes 6–9). Un-processed (full-length) Acp36DE runs at ∼122 kDa. (D) Western blot probed with CG11864 antibody. Female RTs dissected at 45 minutes ASM to seminase control (lane 1) or knockdown (lane 2) males. Numbers to the left of blots indicate approximate band size in kDa.

We tested five predicted protease SFPs for ovulin processing defects via Western blot to identify potential CG11864-interacting proteins. The tested SFPs were the predicted serine proteases ‘seminase’ (CG10586), CG10587, CG4815, CG12558, and CG32382 (sphinx2). Of these, only seminase, a predicted trypsin-type serine protease, was required for ovulin processing (Figure 1A). In addition to the results for seminase, we show for comparison the results for CG10587, which did not affect ovulin processing. The data for the other SFPs tested are not shown. Two independent insertion lines of the same RNAi construct were used to test the phenotype of seminase knockdown (see Materials and Methods); we obtained similar results with both lines. Western blotting confirmed that seminase is knocked down at least 98% by Tubulin-Gal4 driven expression of the RNAi construct in males of both lines (Figure S1A). Transcript levels of *seminase* were also confirmed to be knocked down by RT-PCR (Figure S1B).

Similar to the phenotype previously observed with CG11864 RNAi [32], some ovulin processing was observed in females mated to males knocked down for seminase, but ovulin was never processed fully in mates of seminase knockdown males, even at 2 hours ASM, the latest time at which ovulin can be reliably detected in female reproductive tracts when mated to controls (Figure 1A). Females mated to seminase knockdown males also failed to fully process Acp36DE at 1 hour ASM (Figure 1C), similar to CG11864 knockdown mates (Figure 1C). Even at 3 hours ASM, Acp36DE in females mated to seminase or CG11864 RNAi males had undergone only a small amount of processing relative to controls (Figure 1C).

### Seminase Is Required for Putative CG11864 Pro-Peptide Cleavage

Females mated to seminase RNAi knockdown males received CG11864 protein, but it was of the full-length molecular weight (33-kDa); the cleaved form (30-kDa) was never observed (Figure 1D). However, females who mated to control males received both full-length and cleaved CG11864 (Figure 1D). Thus, seminase is required for the predicted pro-peptide cleavage of CG11864 during mating.

### Seminase Is Specific to the Male Accessory Glands and Is Initially Cleaved in the Male during Transfer to the Female

**Figure 2:**
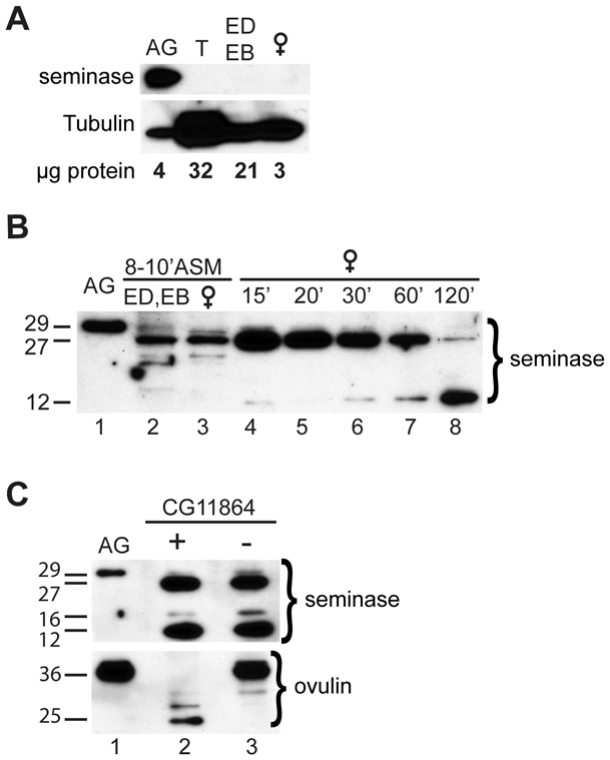
Seminase is produced in the accessory glands and is processed during and after mating. (A) Western blot probed with seminase antibody. Seminase in the male accessory glands (AG) has an apparent molecular weight of ∼29-kDa. No seminase was detected in testes (T), the ejaculatory duct and bulb (ED/EB), or in virgin female reproductive tracts. AG and T: tissue from 10 virgin males. ED/EB: tissue from 20 virgin males. Female: tissue from 4 virgin females. Tubulin is shown as a loading control. Total protein loaded is shown in micrograms as measured by BCA assay. (B) Western blot probed with seminase antibody. Lane 1: full-length seminase in AG. Lane 2: ED/EB dissected from 20 males at 8–10 minutes ASM. Lane 3: Reproductive tracts (RT) from 20 females dissected 8–10 minutes ASM. Lanes 4–8: Female RTs dissected at the times ASM indicated above the lanes. (C) Western blots probed for seminase (top) and ovulin (bottom). Lane 1: full length protein in AG. Lanes 2 and 3: RT from females mated to CG11864 control (+) or knockdown (−) males at 1 hour ASM.

Since many serine proteases are synthesized as zymogens (containing an N-terminal sequence that must be removed for activation), we tested whether seminase was also processed during or after mating. Seminase is detected as an apparent 29-kDa protein (predicted size: 28.2-kDa, excluding a predicted N-term secretion signal sequence) in the accessory glands, with no detectable expression in the testes or ejaculatory duct and bulb (Figure 2A). There was no evidence for seminase expression outside of the male accessory glands based on expression data in the FlyAtlas database [51] and our own RT-PCR (Figure S1B). We did not detect seminase protein in virgin females (Figure 2A).

During mating, an additional, lower molecular weight band (approximately 27-kDa) of seminase appeared in the male ejaculatory duct and/or bulb (Figure 2B), consistent with removal of a 2.79-kDa pro-peptide (size prediction based on an NCBI conserved domain search at http://www.ncbi.nlm.nih.gov/Structure/cdd/wrpsb.cgi?INPUT_TYPE=live&SEQUENCE=NP_649270.1). Within the mated female, seminase was further cleaved, producing a ∼16-kDa product (visible in top panel of Figure 1C) and increasing the amount of the ∼12-kDa product (Figure 2B). We first detected the ∼12-kDa product in the female at around 15 minutes ASM. However, due to the difficulty in detecting the ∼16-kDa form, we could not determine at what time ASM it is first produced.

Given that some proteases can be cleaved by their own proteolytic substrates [for example, see 52], we tested whether knockdown of CG11864 affected processing of seminase after mating. Females mated to CG11864 knockdown males showed normal processing of seminase at 30 minutes ASM (Figure 2C, top panel). As expected [32], CG11864 knockdown does prevent ovulin cleavage (Figure 2C, bottom panel), indicating a unidirectional proteolytic pathway.

### Seminase Is Also Required for CG11864-Independent Post-Mating Processes

**Figure 3:**
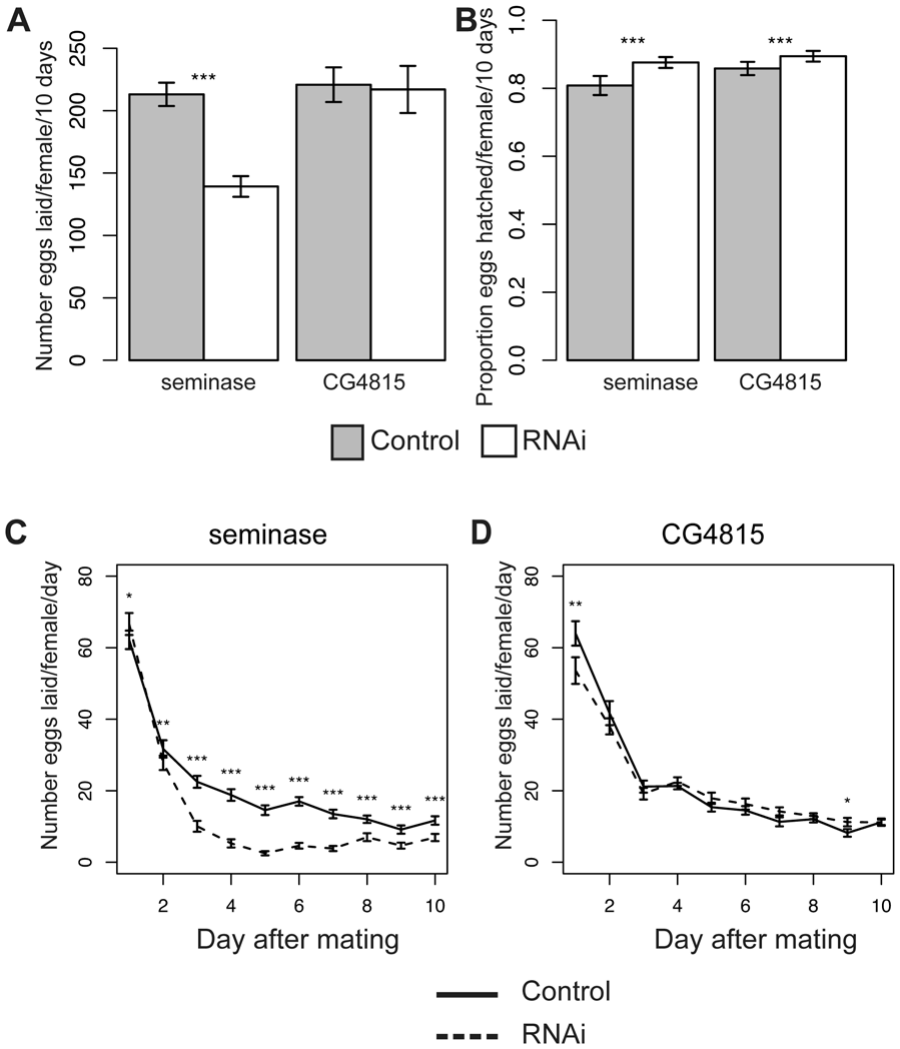
Females mated to seminase knockdown males lay fewer eggs. (A) The average number of eggs laid per female in a given treatment over 10 days. Seminase Line 1: Control N = 55, RNAi N = 59; CG4815: Control N = 20, RNAi N = 18. (B) Hatchability data for the corresponding experiments in (A). Hatchability is defined as the proportion of eggs that yielded adult progeny. CG4815 (a serine protease SFP) is included as a control for strain background. (A),(B) Asterisks indicate p<0.0001. (C) The data from (A) plotted as average number of eggs laid by females in each group on individual days of the experiment. (D) CG4815 egg laying data plotted as in (C). Asterisks indicate level of significance after Bonferroni correction (*p<0.05, **p<0.01, ***p<0.0001). Error bars indicate standard error of the raw data pooled over experiments.

The total number of eggs laid over 10 days was significantly lower in females mated to seminase knockdown males relative to their controls, and this was seen in both independent insertion lines (Line 1 Poisson regression: z = −29.56, p<0.0001, Figure 3A; Line 2 Poisson regression: z = −31.59, p<0.0001, Figure S1A). There was no difference in total number of eggs laid between females mated to CG4815 knockdown and control males (CG4815 Poisson regression: z = −0.79, p = 0.43, Figure 3A).

Eggs laid by seminase or CG4815 knockdown mates hatched in significantly larger proportions than eggs laid by control mates (Binomial regressions: Line 1: z = 10.1, p<0.001 and CG4815: z = 5.8, p<0.0001, Figure 3B; Line 2: z = 13.2, p<0.0001, Figure S1B), suggesting the egg laying defect was not accompanied by a hatchability defect (hatchability is defined as the proportion of eggs that produced adult progeny), but rather that there was a slight deleterious effect of the balancer control background on hatchability.

A repeated measures analysis of egg laying over time revealed a significant effect of male genotype on egg laying over time for both seminase lines and CG4815 (see Materials and Methods). To determine the days on which male genotype affected egg laying, data were analyzed separately for each individual day. The decrease in egg laying, relative to control, in mates of seminase knockdown males was only apparent after the first day following mating and persisted until at least 9 days post-mating (Figure 3C and Figure S1C). Females mated to seminase knockdown males laid slightly, though significantly, more eggs than females mated to control males on day 1, but only with seminase Line 1 males (Figure 3C). These results indicated that seminase only has a major role in egg laying after the first day post-mating.

Females mated to CG4815 knockdown males laid significantly fewer eggs than females mated to control males on day 1 and slightly, though significantly, more eggs than controls on day 9 (Figure 3D). Thus, CG4815 may have a short-term effect on egg laying, but no long-term effect similar to that observed with seminase knockdown mates. These results also indicate that the long-term egg laying effect of seminase is not an artifact of the VDRC strain background, which is shared by the CG4815 males.

A reduction in fecundity after the second day post-mating suggests seminase is a new member of the LTR pathway. Increased recovery of post-mating receptivity to courtship beginning after the first 24 hours post-mating is also associated with LTR defects. Therefore, we tested whether deficiency of seminase in the ejaculate also caused increased receptivity in females, relative to controls, after mating. Table 1 shows data for female receptivity at 24 hours, 2 days, and 4 days ASM to seminase knockdown or control males. Similar to previously described phenotypes of LTR SFPs [36], [42], [43], females mated to seminase knockdown males were significantly more likely to remate at 2 days and 4 days ASM than were controls. Females mated to males from seminase knockdown line 1 showed a smaller magnitude of difference in remating rate relative to their controls than did line 2. This is most likely due to a background effect in line 1 that is apparent in the control males, as the remating rate is similar in mates to knockdown males from both lines. Since the same RNAi construct is expressed in both lines, we assume that the higher remating rate for females mated to line 1 control males is due to the insertion locus of the transgene. There was no effect of CG4815 knockdown on receptivity (Table 1).

**Table 1:** Female receptivity to second mating.

Line	Time ASM	% Control Remated (N)	% RNAi Remated (N)	Chi Square	p value
seminase-1	24 h	0% (29)	0% (25)	NA	NA
seminase-1	2 d	5% (40)	57% (47)	24.44	**7.67E-07**
seminase-1	4 d	42% (31)	94% (32)	17.19	**3.38E-05**
seminase-2	24 h	0% (28)	3.3% (30)	0.001	0.972
seminase-2	2 d	2% (47)	30% (46)	11.76	**6.06E-04**
seminase-2	4 d	1.7% (57)	90% (52)	83.11	**7.74E-20**
CG4815	4 d	0 (20)	0 (19)	NA	NA

Females were mated first to control or RNAi knockdown males from the line shown (either seminase line 1 or 2, or CG4815). CG4815 is included as a control for the VDRC strain background. The time given is the number of days after the first mating that the second mating was attempted. Females who remated to a male from a wildtype strain (Canton-S) during a one hour observation period are shown as a percentage with the total number of potential mating pairs assayed in brackets. P values in bold are significant.

### Seminase Is Required for Sperm Release from Storage

**Figure 4:**
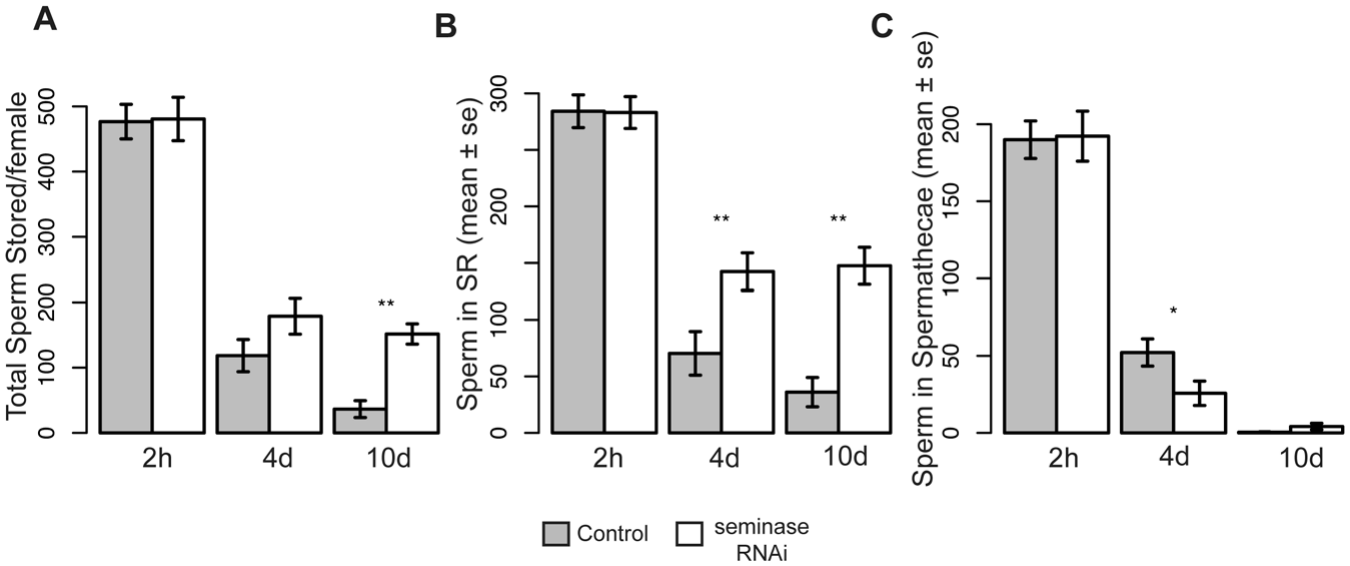
Females mated to seminase knockdown males retain more sperm 4 and 10 days after mating. Sperm counts for seminase line 1 are shown for sperm stored at three timepoints after mating, as given on the x-axis. Line 2 yielded similar results. Asterisks indicate level of significance (*p<0.05; **p<0.01). (A) Total average number of sperm stored in both storage organs (2 h: t = −0.09, p = 0.93; 4 d: t = −1.6, p = 0.13; 10 d: t = −3.2, p<0.01). (B) Average number of sperm stored in the seminal receptacle only (2 h: t = −0.06, p = 0.96; 4 d: t = −2.8, p<0.01; 10 d: t = −3.2, p<0.01). (C) Average number of sperm stored in the paired spermathecae only; numbers are the sum of sperm stored in each spermatheca (2 h: t = −0.11, p = 0.91; 4 d: t = 2.2, p<0.05; 10 d: t = −1.1, p = 0.26). Samples sizes are, for bars from left to right: (A) 12, 14, 16, 10, 7, 15; (B) 17, 21, 18, 19, 7, 15; (C) 13, 14, 19, 10, 7, 15. Abbreviations: 2 h, 2 hours; 4 d, four days; 10 d, 10 days. Error bars indicate standard error.

In *Drosophila* females, sperm are stored in two types of storage organs: the seminal receptacle (SR) and the paired spermathecae. The bulk of the sperm are stored in the SR [53]. Because LTR SFPs (in concert with SP) affect the release of sperm from storage [36],[43], we tested whether mates of seminase knockdown males also showed a defect in sperm release. Mates to seminase knockdown males stored normal numbers of sperm (Figure 4 “2 h” bars), but significantly more sperm remained in storage at 10 days ASM in mates of seminase knockdown males than in control-mated females (Figure 4A). This effect was due to a failure to release sperm from the SR (Figure 4B), as sperm numbers in the spermathecae decreased at similar rates in females mated both to control and seminase knockdown males (Figure 4C). A slight, but significant, difference in sperm release was seen in the spermathecae at 4 days ASM, but this effect was in the opposite direction from that seen in the SR and was no longer apparent by 10 days ASM (Figure 4C). Similar effects were seen with seminase knockdown Line 2 (Figure S1D).

### SP and Other LTR Proteins Fail to Localize to the Seminal Receptacle in the Absence of Seminase

**Figure 5:**
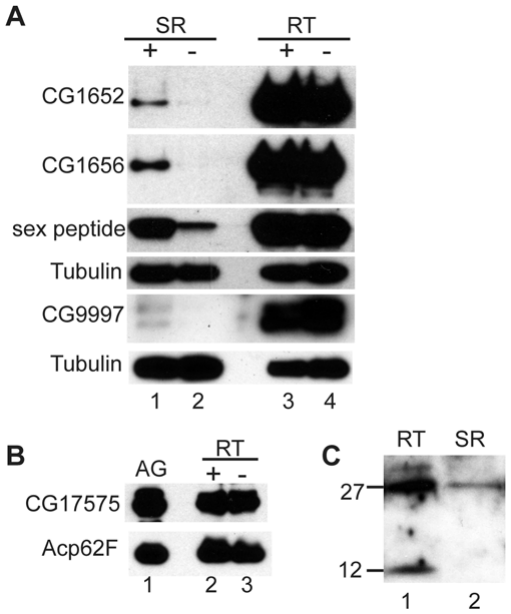
LTR proteins fail to accumulate in the seminal receptacle in the absence of seminase. (A) Western blots probed for the proteins indicated to the left of blots. Lanes 1 and 2 contain 20 seminal receptacles (SR) dissected from females mated to control (+) or seminase knockdown (−) males at 2 hours ASM for SP, CG1656, and CG1652, and 1 hour ASM for CG9997. CG9997 was probed on a separate blot from CG1652, CG1656, and sex peptide. Lanes 4 and 5 contain 4 reproductive tracts (RT; not including ovaries or sperm storage organs, SSOs) from a subset of the females dissected in SR lanes. Tubulin is shown as a loading control for both gels. (B) Western blot probed for CG17575. Lane 1: male accessory glands (AG). Lanes 2 and 3 contain 4 RTs (excluding ovaries) from females mated to control (+) or seminase knockdown (−) males at 45 minutes ASM. Acp62F is shown as a loading control. (C) Western blot probed for seminase. Lane 1 contains female RT excluding ovaries and SSOs from wildtype mated females. Lane 2 contains 80 SR from wildtype mated females.

The results above are consistent with seminase being a member of the LTR network. To determine the placement of seminase in this network, we tested whether knockdown of seminase affected the post-mating localization of SP and the three LTR proteins that localize to the SR: CG9997, CG1652, and CG1656. At 2 hours ASM, seminase was required for accumulation of SP, CG1652, and CG1656 in the SR (Figure 5A). CG9997 was not detected in the SR at 2 hours ASM, so we tested females at 1 hour ASM. Seminase was also required for accumulation of CG9997 in the SR at this time point (Figure 5A). However, seminase was not required for proper processing of CG9997 or transfer of any LTR SFPs to the female during mating (Figure 5A, RT lanes), including CG17575 (Figure 5B).

A small amount of seminase also enters the SR (Figure 5C), suggesting that it could physically interact with other LTR proteins there. However, we were unable to determine whether other LTR proteins affected seminase localization to the SR due to the extremely low seminase signal within the SR. Multiple repetitions of the experiment failed to yield consistent results. As with previous efforts to detect LTR proteins in the spermathecae [42], we were not able to detect seminase in these organs (data not shown).

## Discussion

### An SFP, the Predicted Serine Protease Seminase, Regulates Processing of Other SFPs through a Proteolytic Cascade

To identify proteins that may interact with the predicted astacin-family protease CG11864 to process the SFPs ovulin and Acp36DE, we used RNAi to individually test five serine protease SFPs for ovulin processing defects. One of these proteins, the predicted trypsin-type serine protease ‘seminase’ (CG10586), is required for normal processing of ovulin as well as of the sperm storage protein Acp36DE. Because the phenotype of seminase knockdown was similar to that of CG11864 knockdown with respect to SFP processing, we hypothesized that both proteins might act in a single pathway. Additionally, because trypsin (serine) proteases are required for activational cleavage of some astacin-family proteases (of which CG11864 is one) [50], [54], we further hypothesized that seminase might act upstream of CG11864.

We therefore tested whether seminase regulates the cleavage, and thus activation, of CG11864. We found that seminase is required for the approximately 3-kDa mobility shift of CG11864 that is seen in the male reproductive tract very soon after mating begins, suggesting that seminase may activate CG11864 by cleaving its pro-peptide. The apparent processing of pro-CG11864 by seminase and the subsequent processing of downstream substrates is suggestive of a proteolytic cascade. No such proteolytic pathway has, to our knowledge, previously been identified in insect seminal fluid. With the identification of this pathway in *Drosophila melanogaster*, we have found a molecular model for dissecting proteolytic pathways involving SFPs that have consequences for fertility.

Proteolytic cascades, in their simplest form, typically have three steps [55]: 1) auto-activation of an initiator protease(s) present in low amounts and triggered by an external stimulus: 2) activation of a more abundant propagator protease(s) by the initiator protease; 3) activation of an executor protease(s) by the propagator, which will cleave the downstream substrates. In addition, the propagator may also cleave, and thereby continue to activate, the initiator. Altogether, this causes a rapid propagation of the initial external signal.

While protein abundance is not necessarily related to potency, it is intriguing that seminase (the putative initiator) is relatively scarce in the ejaculate of *D. melanogaster*, which is consistent with the above model. Abundance estimates are based on the normalized spectral abundance factor (NSAF) obtained by mass spectrometry on mated females [2]. NSAF is an approximate measure of the relative abundance of a protein in a complex sample. Seminase ranks at 130 out of 138 (NSAF = 1.34×10^−4^), with 1 being the most abundant and 138 the least [2]. CG11864 is similarly scarce (87/138; NSAF = 7.69×10×^−4^). This is in contrast to the much higher abundance of CG11864's substrates (ovulin: 20/138, NSAF = 1.2×10^−2^; Acp36DE: 19/138, NSAF = 1.21×10^−2^).

Also consistent with the protease cascade model, seminase is cleaved to a slightly smaller form during mating while still in the male reproductive tract. This may be an activational pro-peptide cleavage event, though this has not been directly tested. It is possible that seminase self-activates upon entering the ejaculatory duct, as is the case for many serine proteases [for example, among tissue kallikrein pathways; see 15]. Our data suggest that seminase acts as the initiator in the cascade, and CG11864 acts either as the propagator, the executor, or both.

After transfer, seminase itself undergoes additional processing in the female (after the initial pro-peptide cleavage in the male) that is not a result of CG11864 activity (Figure 2C). These cleavage products may be important for the function of seminase. On the other hand, they may simply be degradation products of seminase. However, both scenarios remain speculative.

We have shown that, in the absence of seminase, CG11864 is not cleaved to the predicted active form. However, the predicted pro-peptide cleavage site of CG11864, based on sequence threading to other astacin-family proteases [32], is not a trypsin site, as would be predicted if seminase were the only protease responsible for CG11864 activation. Interestingly, there are three trypsin cleavage sites present in the pro-peptide region of CG11864. It is possible that CG11864 is cleaved via a two-step mechanism (involving a trypsin and CG11864 itself), as is seen for the pro-peptide cleavage of *Astacus astacus* (crayfish) astacin, the prototype of the astacin family [54], [56]. Future studies using purified proteins *in vitro* will determine whether CG11864 is capable of self-cleavage and whether seminase acts to directly cleave CG11864.

### Role of the Seminase/CG11864 Pathway

Despite a severe delay in ovulin processing, knockdown of neither seminase nor CG11864 results in an egg laying defect in the first 24 hours after mating [CG11864 data reported in 32]. This result is not surprising, however, given the rather small effect on egg laying seen with a complete knockout of ovulin [45]. Additionally, ectopic expression of full-length ovulin is sufficient to induce ovulation in virgin females [46], suggesting that the additional effect of ovulin processing may be too small to detect with the current assay. It is also possible that, while seminase was knocked down to very low levels, there may still be sufficient seminase present for its role in early egg laying.

We also do not observe a defect in sperm entry into storage following seminase knockdown, as seen with knockout of Acp36DE [37]. Instead, defects were seen in sperm release from storage at later timepoints, which is not a phenotype associated with Acp36DE knockout. Acp36DE processing may be important for other functions of this protein. For example, Acp36DE is a component of the mating plug [57], but its function in the mating plug is still unknown. Further research is required to determine the consequences of loss of proteolytic processing of both ovulin and Acp36DE.

### Seminase Regulates Multiple Independent Post-Mating Processes

**Figure 6:**
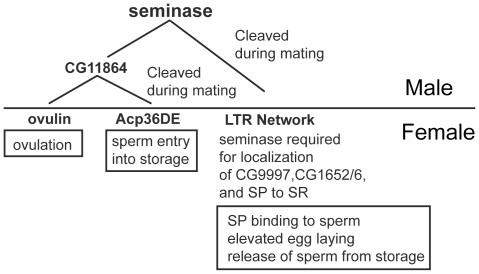
Seminase is required for two post-mating pathways. Seminase is cleaved during mating within the male reproductive tract where it is required for CG11864 cleavage. CG11864 then regulates processing of two SFPs, ovulin and Acp36DE (left branch). Seminase is also necessary for the long term response (LTR) pathway (right branch). Proteins/pathways downstream of seminase are involved in the processes shown in boxes, based on earlier knockout or knockdown studies. The consequences of proteolytic processing of ovulin and Acp36DE remain unknown.

In contrast to CG11864, which seems specific to the STR, seminase has a second important activity: it is in the LTR pathway, which regulates the binding of sex peptide (SP) to sperm [36], [41]–[43] (See Figure 6 for overview). Similar to mates of SP null males, females mated to seminase knockdown males lay fewer eggs than controls over a 10 day period and also retain sperm in storage. The sperm retention phenotype is only apparent in the SR. Other LTR proteins are also known to affect sperm storage in the SR but not the spermathecae [36], though the reason for these differences is not understood. While the interaction between sperm release and egg laying is complex, over the long-term, egg laying and sperm release are independent of each other [58]. Sperm do not directly influence the release of eggs, though the presence of SP bound to sperm is required for both sperm release [43] and normal post-mating levels of egg laying [39], [40]. The failure of SP to accumulate in the SR indicates that seminase is likely required for SP to bind sperm.

The requirement for seminase in two independent post-mating pathways suggests that its activation at mating may act as a regulatory “switch” that coordinates post-mating events in *Drosophila*. Identification of other seminase substrates, if they exist, will allow us to determine the extent of seminase's effects as a regulatory switch for post-mating events.

### Evolutionary Implications of Seminase-Regulated Processes

In the context of evolution, SFPs represent a unique class of proteins in that they must, first and foremost, aid in successful fertilization, but are also tasked with representing the male's reproductive interests, sometimes in the face of opposing female interests [reviewed in 59]. This has the potential to set up a genetic conflict between the sexes and has been suggested to be one reason that SFPs in particular tend to be rapidly evolving [24], [25]. However, an SFP's evolutionary rate is also likely to be constrained by the need for the protein to maintain its interaction with other proteins in the seminal fluid, and/or with proteins expressed by the female. For example, seminase and CG11864 are processed first in the male, but must interact with the female environment to further process both seminase and the substrates of the proteolytic pathway. Seminase-regulated processes represent an opportunity to understand the evolution of SFP networks that contain a mixture of conserved proteins (*e.g.* the lectins CG1652 and CG1656 and the CRISP CG17575 [2]) and proteins under positive selection (*e.g.* ovulin [60] and the serine protease CG9997 [61]). Seminase itself shows no evidence of positive selection, either at the protein level [61] or at individual sites (personal communication, Geoff Findlay). However, seminase does have two very closely related SFP paralogs, CG11037 and CG10587, which show evidence for recent positive selection in the *D. melanogaster* lineage [61]. These three genes are clustered together in the genome of *D. melanogaster* and the other *melanogaster* subgroup species. CG10587 does not play a role in ovulin or Acp36DE processing (CG11037 has yet to be tested), suggesting that these genes arose from tandem duplications and later diverged in function, with seminase remaining as the more conserved of the paralogs.

Our data on seminase show that this member of a conserved protein class in the seminal fluid plays a vital role in reproductive success. We believe that future study of the seminase-regulated pathways in *Drosophila* will lead to new mechanistic and evolutionary insights related to proteolytic cascades and protein networks in seminal fluid.

### Seminase-Dependent Processes: A Model for Pleiotropic SFPs

The regulation and mechanism of action of seminase constitutes a new *in vivo* model system for studying the regulation and physiological roles of pleiotropic SFPs. Pleiotropic effects of kallikrein-related proteases involved in the liquefaction of human semen have recently been reported [62], [63]. Further understanding of the various effects of seminal fluid proteolysis in post-mating processes may have important implications for human health (*e.g.* the role of PSA in cancer) and fertility. Our results indicate that genetic analysis in *Drosophila* will be an important complement to *in vitro* studies in mammalian systems for understanding the role of proteolytic processing in reproduction. Future studies of the regulatory mechanisms involved in the seminase/CG11864 proteolytic pathway may generate testable hypotheses for other SFP networks, including those in mammals.

## Materials and Methods

### Flies

Transgenic lines carrying RNAi constructs for *CG10586 (‘seminase’)* and *CG4815* were purchased from the Vienna Drosophila Resource Center (VDRC, http://stockcenter.vdrc.at) GD RNAi library (P element library). VDRC lines used correspond to the following transformant ID numbers: 18795, 18796 (*CG10586*; same construct (ID 5539), different insertions), and 15410 (*CG4815*). *CG10586*-18795 is referred to here as “Line 1” and *CG10586*-18796 as “Line 2”. *CG10586* VDRC lines are predicted to have an off-target, *CG33306*. However, we found no evidence for *CG33306* knockdown in either line (Figure S2B, only Line 1 shown). All RNAi knockdown and control sibling flies were produced by crossing sympUAST-*SFP* or VDRC virgin females to ubiquitous driver males (*Tubulin-Gal4/ TM3*). *UAS-RNAi / Tubulin-Gal4* (non-balancer) male progeny were knocked down for the SFP of interest and the sibling *UAS-RNAi/TM3* (balancer) flies were used as controls that are wildtype for seminase expression.

Matings were carried out by placing single 3–6 day old virgin females of the Canton-S strain with a single 3–6 day old virgin male in a glass vial containing a moistened square of filter paper. Matings were observed and the time at which mating began was recorded. Mating pairs with unusually short matings (<15 minutes) were discarded. Mated females were flash frozen in liquid nitrogen at the appropriate time after the start of mating (ASM) for time points less than one hour ASM and stored at −80°C until dissection. All flies were reared in standard yeast-glucose media at room temperature (23±1°C) on a 12∶12 light/dark cycle.

### Seminase Antibody Production

Generation of seminase fusion proteins and antibody purification was done following Ravi Ram et al. [64] and Cui et al. [65]. Briefly, we generated a 6×His fusion protein containing amino acids 101–200 from seminase-PA using the pDEST17 vector of the Gateway system (Invitrogen). Antibodies were generated in rabbits (Cocalico) as described previously for eight other *Drosophila* reproductive proteins, including CG11864 [64], and Wisp [65] except that rabbits were immunized with the 6×His-seminase fusion protein. Anti-seminase was affinity purified with a GST fusion protein of amino acids 101–200 of seminase, as described in the above references. Eluted antibodies were stored at −20°C in glycerol (1∶1), and used at a concentration of 1∶250 for Western blot analysis.

### Sample Preparation and Western Blot Analysis

Sample preparation and Western blot analyses in Figure 1A and Figure 5A and 5B were carried out as in Ravi Ram and Wolfner [42]. Samples in other Westerns in this study were prepared similarly, except that they were separated using 5–15% gradient SDS/PAGE. Female reproductive tract samples (RT) are lower reproductive tract extracts (ovaries removed) from 4–6 mated females, unless otherwise noted.

A BCA (bicinchoninic acid) assay (Pierce BCA Protein Assay Kit, Thermo Scientific) was performed to determine the protein loading in Figure 1A. Samples identical to those used for the Western blot were prepared and protein concentration measured relative to a BSA standard, following the manufacturer's guidelines.

### Fecundity/Fertility Assays

Number of eggs laid daily by mated females (fecundity) and number of progeny produced from those eggs (fertility) were quantified as in Ravi Ram and Wolfner [36]. Assays for the effect of seminase on fecundity and fertility were carried out three times for each independent insertion line, each time with 15–24 females measured for both knock down and control treatments. CG4815 knock down- and control-mated females were also measured for both fertility and fecundity, as a control for the VDRC background. Two assays were carried out with this line, each time with 7–12 females measured for each treatment. Hatchability was determined by dividing number of progeny by number of eggs (fertility/fecundity) [36]. Inspection of the data revealed non-significant variation in egg laying due to experimental block, so data were pooled across blocks. The effect of seminase or CG4815 knockdown on total 10-day egg laying was tested with a Poisson regression model (using the R function ‘glm()’. The statistical tests for hatchability were the same, except a Binomial regression was used.

A repeated measures analysis was performed to determine the effect of male genotype over time. This analysis was performed using a Poisson mixed-effects model with the R function ‘lmer()’ in the lme4 library. Two models were compared, a full model with day, genotype, and day-by-genotype interaction as fixed effects and female as the random effect, and a model with day as the only fixed effect. Comparison of the two models by ANOVA (R function ‘anova()’) revealed the full model was the better fit, indicating a significant effect of male genotype. To determine the statistical significance of male treatment on individual days post-mating, a Bonferroni correction for multiple tests was applied to the Poisson regressions. All plots were generated using the means and standard errors of the raw data pooled from all experiments. Statistical significance of the effect of male genotype on number of eggs laid is denoted by asterisks on the plots.

### Receptivity Assays

Females who had previously mated with either control or seminase knockdown males were placed with a single wildtype male of the Canton-S strain for one hour either 24 hours, 2 days, or 4 days following the initial mating and the number of copulations beginning within one hour was recorded. Receptivity response to remating was tested for seminase as in Ravi Ram and Wolfner [36]. No fewer than 10 females were analyzed for control and experimental groups at 24 hours, 2 days, or 4 days after initial mating. Data were analyzed using a Chi-squared test (R function ‘chisq.test()’ with all parameters set to default).

### Sperm Counts

Sperm counts were performed as in Avila, *et al.* [43] at 2 h ASM, 4 days, and 10 days ASM. Sample identity was coded to avoid bias and each slide was counted twice to assess counting precision (85%–100%). SR data used are the average of the two counts. Spermathecae data are the sum of the two averages (one for each spermatheca). For 4 and 10 day post-mating samples, individual female daily egg counts were also taken. Numbers of stored sperm at 4 and 10 days ASM were significantly negatively correlated with the number of eggs laid (Figure S2). Females that laid very few eggs on day 1 (less than 2 standard deviations below the mean) were removed from the dataset as they were likely unhealthy and may have had improper sperm storage. Data were analyzed using a two-tailed Student's t-test (R function ‘t.test()’).

## Supporting Information

Figure S1 Egg laying, hatchability, and sperm storage in females mated to seminase Line 2 males. (A) The average number of eggs laid per female in a given treatment over 10 days in seminase (Control N = 40, RNAi N = 49). (B) Hatchability data for the experiment in (A). (A),(B) Asterisks indicate p<0.0001. (C) The data from (A) plotted as average number of eggs laid by females in each group on individual days of the experiment. Asterisks indicate level of significance after Bonferroni correction (*p<0.05, **p<0.01, ***p<0.0001). (D) Sperm storage results for seminase Line 2, plotted as in Figure 4. Left panel: Total average number of sperm stored in both storage organs (2 h: t = −1.45, p = 0.15; 4 d: t = −2.5, p<0.05; 10 d: t = −4.4, p<0.001). Middle panel: Average number of sperm stored in the seminal receptacle only (2 h: t = −1.6, p = 0.12; 4 d: t = −3.8, p<0.001; 10 d: t = −4.9, p<0.0001). Right panel: Average number of sperm stored in the paired spermathecae only; numbers are the sum of sperm stored in each spermatheca (2 h: t = −2.02, p = 0.053; 4 d: t = 2.62, p<0.05; 10 d: t = −0.23, p = 0.82). Asterisks indicate level of significance. Error bars indicate standard error. Sample sizes are shown above each bar.
(TIF)

Figure S2 Levels of seminase protein are reduced in RNAi lines. (A) Western blot probed for seminase. Both independent insertion lines for the seminase RNAi construct are shown (Line 1 and line 2). Bands not affected by knockdown are assumed to be nonspecific cross-reactants. Arrow points to the seminase band. Each lane contains proteins from male accessory glands (AG). Lanes marked “100%” contain AG protiens from 10 males, “50%” from 5 males, “10%: from 1 male, and “2%” from the equivalent of 1/5 male. The bottom panel is the same Western blot probed for CG11864 as a loading control. Lanes 1–4: AG from line 1 control males. Lanes 7–10: AG from line 2 control males. Lanes 5–6: AG from line 1 RNAi males. Lanes 11–12: AG from line 2 RNAi males. (B) RT-PCR testing for presence of full-length seminase transcript (top panel) and a 200 bp fragment of the CG33306 transcript (bottom panel). CG33306 is a potential off-target of seminase knockdown as predicted by the VDRC. Lanes 1–3 are from knockdown (KD) males. Lanes 4–6 are from sibling control males. Only results for Line 1 are shown, though similar results were obtained for Line 2. T: testes; C: carcass (whole male minus reproductive tract).
(TIF)
