# Supplementary figures and images for: The Drosophila melanogaster Seminal Fluid Protease “Seminase” Regulates Proteolytic and Post-Mating Reproductive Processes
Source: PLoS Genet. 2012 Jan 12;8(1):e1002435. doi: 10.1371/journal.pgen.1002435 (PMC3257295; doi:10.1371/journal.pgen.1002435)

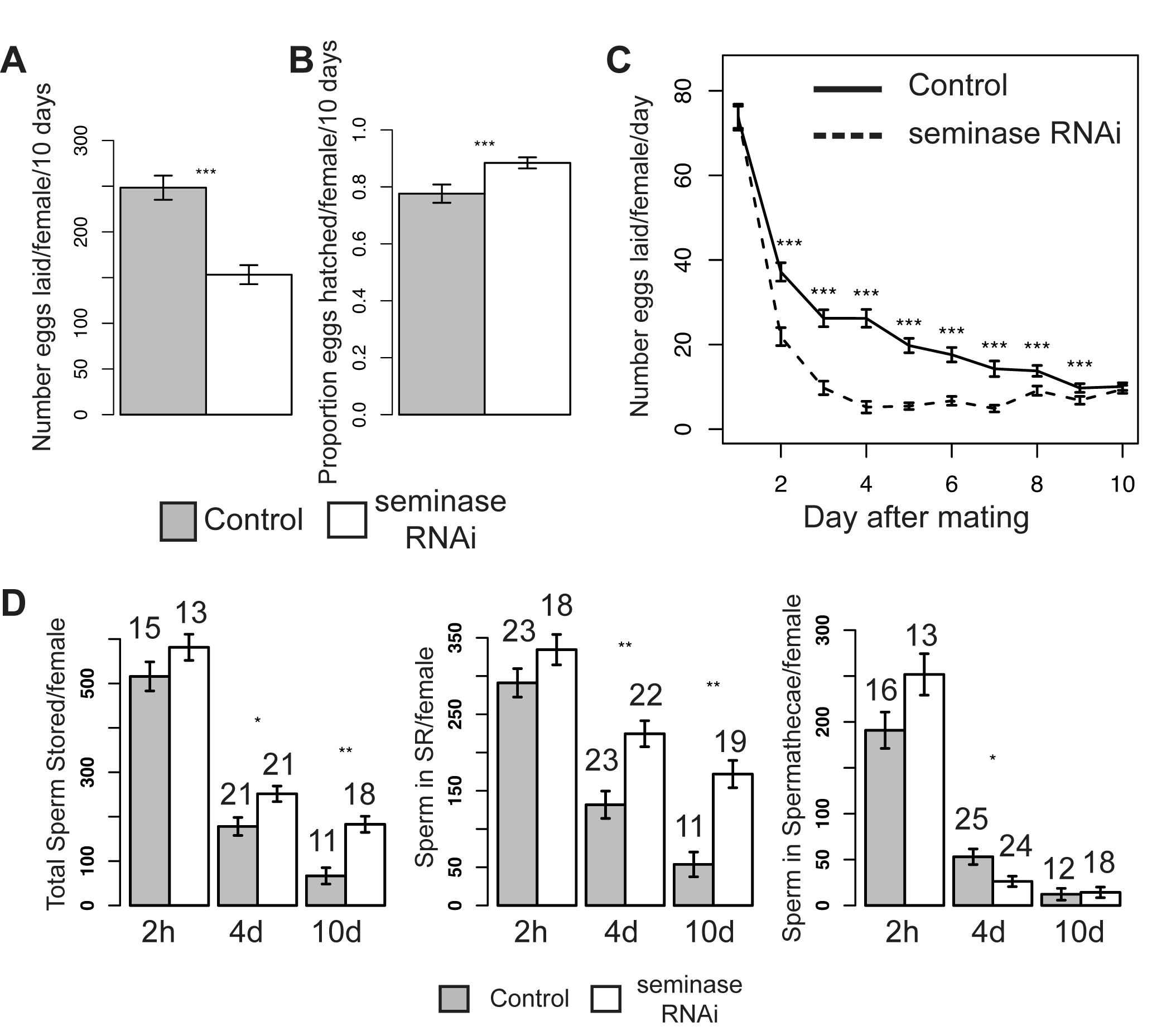

Supplement: Figure S1 — Egg laying, hatchability, and sperm storage in females mated to seminase Line 2 males. (A) The average number of eggs laid per female in a given treatment over 10 days in seminase (Control N = 40, RNAi N = 49). (B) Hatchability data for the experiment in (A). (A),(B) Asterisks indicate p<0.0001. (C) The data from (A) plotted as average number of eggs laid by females in each group on individual days of the experiment. Asterisks indicate level of significance after Bonferroni correction (*p<0.05, **p<0.01, ***p<0.0001). (D) Sperm storage results for seminase Line 2, plotted as in Figure 4. Left panel: Total average number of sperm stored in both storage organs (2 h: t = −1.45, p = 0.15; 4 d: t = −2.5, p<0.05; 10 d: t = −4.4, p<0.001). Middle panel: Average number of sperm stored in the seminal receptacle only (2 h: t = −1.6, p = 0.12; 4 d: t = −3.8, p<0.001; 10 d: t = −4.9, p<0.0001). Right panel: Average number of sperm stored in the paired spermathecae only; numbers are the sum of sperm stored in each spermatheca (2 h: t = −2.02, p = 0.053; 4 d: t = 2.62, p<0.05; 10 d: t = −0.23, p = 0.82). Asterisks indicate level of significance. Error bars indicate standard error. Sample sizes are shown above each bar. (TIF) [file pgen.1002435.s001.tif]

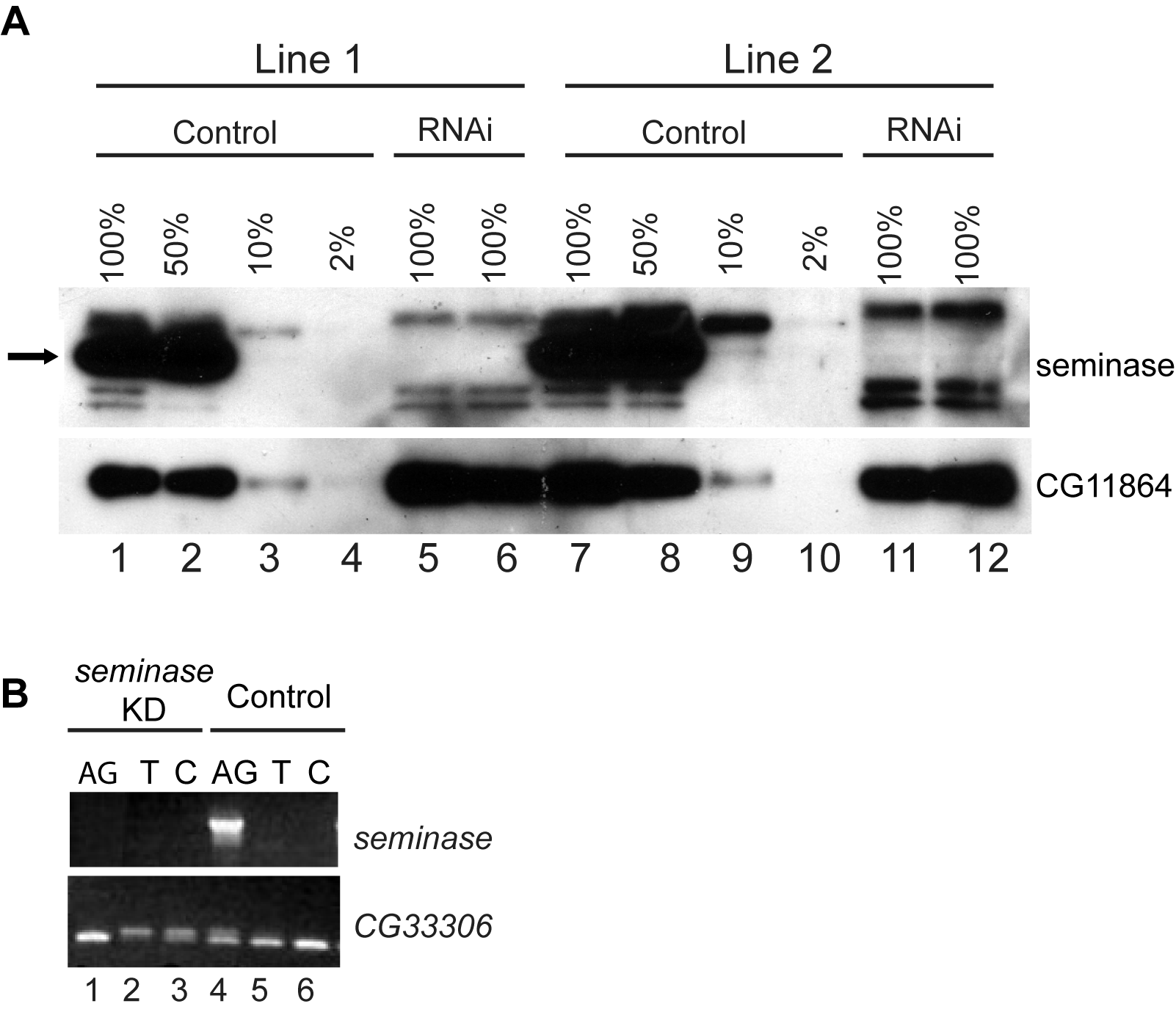

Supplement: Figure S2 — Levels of seminase protein are reduced in RNAi lines. (A) Western blot probed for seminase. Both independent insertion lines for the seminase RNAi construct are shown (Line 1 and line 2). Bands not affected by knockdown are assumed to be nonspecific cross-reactants. Arrow points to the seminase band. Each lane contains proteins from male accessory glands (AG). Lanes marked “100%” contain AG protiens from 10 males, “50%” from 5 males, “10%: from 1 male, and “2%” from the equivalent of 1/5 male. The bottom panel is the same Western blot probed for CG11864 as a loading control. Lanes 1–4: AG from line 1 control males. Lanes 7–10: AG from line 2 control males. Lanes 5–6: AG from line 1 RNAi males. Lanes 11–12: AG from line 2 RNAi males. (B) RT-PCR testing for presence of full-length seminase transcript (top panel) and a 200 bp fragment of the CG33306 transcript (bottom panel). CG33306 is a potential off-target of seminase knockdown as predicted by the VDRC. Lanes 1–3 are from knockdown (KD) males. Lanes 4–6 are from sibling control males. Only results for Line 1 are shown, though similar results were obtained for Line 2. T: testes; C: carcass (whole male minus reproductive tract). (TIF) [file pgen.1002435.s002.tif]
